# Bioremediation of Total Petroleum Hydrocarbons (TPH) by Bioaugmentation and Biostimulation in Water with Floating Oil Spill Containment Booms as Bioreactor Basin

**DOI:** 10.3390/ijerph18052226

**Published:** 2021-02-24

**Authors:** Khalid Sayed, Lavania Baloo, Naresh Kumar Sharma

**Affiliations:** 1 Civil and Environmental Engineering Department, Universiti Teknologi Petronas, Seri Iskandar, Perak 32610, Malaysia; lavania.baloo@utp.edu.my; 2 Kalasalingam Academy of Research and Education, Krishnankoil, Srivilliputhur, Tamil Nadu 626128, India; naresh@klu.ac.in

**Keywords:** oil spill clean-up, oil spill treatment, crude oil, petroleum products, bacteria, fungi, algae, agro-industrial wastes

## Abstract

A crude oil spill is a common issue during offshore oil drilling, transport and transfer to onshore. Second, the production of petroleum refinery effluent is known to cause pollution due to its toxic effluent discharge. Sea habitats and onshore soil biota are affected by total petroleum hydrocarbons (TPH) as a pollutant in their natural environment. Crude oil pollution in seawater, estuaries and beaches requires an efficient process of cleaning. To remove crude oil pollutants from seawater, various physicochemical and biological treatment methods have been applied worldwide. A biological treatment method using bacteria, fungi and algae has recently gained a lot of attention due to its efficiency and lower cost. This review introduces various studies related to the bioremediation of crude oil, TPH and related petroleum products by bioaugmentation and biostimulation or both together. Bioremediation studies mentioned in this paper can be used for treatment such as emulsified residual spilled oil in seawater with floating oil spill containment booms as an enclosed basin such as a bioreactor, for petroleum hydrocarbons as a pollutant that will help environmental researchers solve these problems and completely clean-up oil spills in seawater.

## 1. Introduction 

The world is dominated by five massive oceans and the three main seas, which together account for 71% of the Earth [1]. For thousands of years, the ocean has attracted human attention. It is also the food chain’s principal source and popular for its diverse aquatic species [2,3,4]. Several researchers have warned about the dangers to oceans and acknowledged the threat to human survival by bioaccumulation and biomagnifications of toxic substances in petroleum hydrocarbons [4,5]. There are many forms of life in these oceans, and for this reason specific laws and regulations are continually framed to take care of this insubstantial marine environment. New approaches must, therefore, be developed for managing existing marine ecosystem resources in order to preserve human safety from toxic petroleum hydrocarbons through bioaccumulation and biomagnifications in the food chain [4,6]. 

The largest group of environmental pollutants worldwide is produced from crude oil-based hydrocarbons [7]. Processing activities in the hydrocarbon oil industry releases hazardous aromatic organic compounds such as polyaromatic hydrocarbons (PAHs), phenolic substances that are barely degradable by nature, chlorophenols and cresols toxins from hydrocarbons into the environment [8,9,10]. On the other hand, crude oil spills have intensified oil pollution problems during transportation and storage operations. Crude oil spill in seawater requires an effective clean-up treatment process. Various physicochemical and biological treatment processes have been applied worldwide to eliminate crude oil spill pollution from the seawater. A biological treatment process using bacteria, fungi and algae for biodegradation of crude oil recently received much attention because of its efficiency and lower cost. Researchers have used bioaugmentation i.e., single strain and consortium micro-organisms to degrade the maximum part of the spilled crude oil as a part of the treatment.

Presently, there is a trend towards enhancing and putting back micro-organisms with high potential agro-industrial waste. A variety of low-cost substrates exist such as soybean waste oil, paneer whey, solid-waste-date, corn-steep-liquor, molasses, etc. All these agro-industrial wastes not only serve as nutrients for the growth of organisms, but also act as the main source for potential micro-organisms generating biosurfactants [11,12,13,14,15,16,17]. Some researchers have used other methods by applying N:P:K nutrient ratios [18,19], food wastes [20]. Some researchers have applied it in the field [12,15,18,19,21,22,23,24]. A comprehensive and practical collection of guidelines for the application of this technology to seawater oil spill responders is urgently required to address questions such as when to use bioremediation, what bioremediation agents should be used, how to apply them, and how to track and evaluate the outcomes [25]. The application of micro-organisms for the bioremediation of petroleum hydrocarbon pollutants in this day and age is a priority in the effort to establish green technology [22,23,24,25,26]. The lack of guidelines as to how and when to use this technology is now one of the biggest obstacles for the implementation of oil bioremediation in marine water. One good and beneficial factor is the possibility of using bioremediation methods where water movement is less in an encircled area. This can be done with floating oil containment booms, usually used to contain oil spills in flowing water and restrict moving water movement with oil spills that create an enclosed area, known as the booming technique. Potential studies have been reviewed in this review paper to completely clean-up crude oil spills, TPH and related petroleum products using bioremediation as polishing treatment in combination with floating oil containment booms like a bioreactor basin. The novelty here is that no researcher has used bioremediation in combination with a booming technique that can be used as a bioreactor-like floating oil container basin where micro-organisms can break waste into stable products (carbon dioxide, water etc.).

### 1.1. Petroleum Hydrocarbons

TPH is a term used to represent petroleum (crude oil) that consists of a blend of thousands of compounds. TPH is a chemical combination in this context. They are referred to as hydrocarbons because almost all consist of hydrogen and carbon. Petroleum hydrocarbons account for 50–98% of crude oil and are considered an important component depending on the source of petroleum [27]. The main composition of crude oil is illustrated in Table 1.

**Figure 1:**
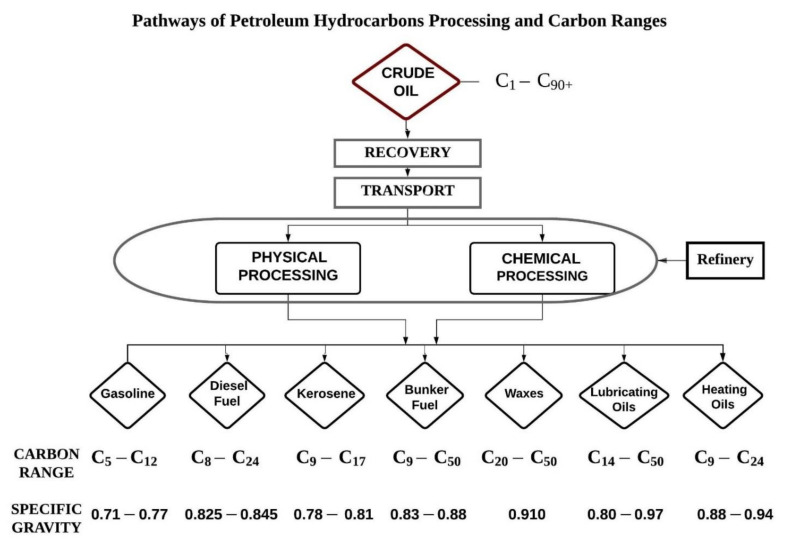
Petroleum hydrocarbon products and fractions by carbon ranges.

Crude oil is extracted from offshore oil rigs in seawater and transported to the shore. Crude oil recovered from the sub surface is of no use directly, for this reason it must undergo refining for a variety of applications. In a petroleum oil refinery crude oil undergoes processes. The oil refinery methods and processes refine products like petrol, gasoline, diesel, jet fuel, asphalt, wax, lubricating oil, tar, kerosene, and liquefied petroleum gas (LPG), etc. The petroleum industry supplies a substantial quantity of world’s energy demands in addition to popular petro-chemical intermediates required for production of extensive range of goods viz. solvents, dye stuffs, pharmaceuticals, polymers, and new chemicals etc. All these goods generate environmental pollution when discharged in the environment [9,28]. Figure 1 show the different products obtained from the petroleum hydrocarbon refinery with their molecular carbon ranges. Carbon ranges illustrated may differ from state to state. These ranges mentioned are the most common.

The stability and behavior of petroleum in seawater depends on its relative density (the relationship between the density of petroleum and pure water) and the distillation characteristics (definition of volatility, large quantities of resin, asphalt, wax, etc. reduces the volatility of oil), viscosity (flow resistance that varies with temperature) and point of pour [29]. Due to the formation of water or gas, or liquids and chemicals extracted during operations, inorganic salts like sodium chloride, magnesium chloride and other inorganic salts often follow crude oil from wells. Heavy crude oils produce large quantities of difficult to process complex hydrocarbons, such as polynuclear aromatic compounds (PNA), PAHs, alkyl aromatic compounds, heteroatoms, and metal materials. Sulphur, oxygen, nitrogen, and metal atoms are typical heteroatoms in hydrocarbons [21].

### 1.2. Sources of Petroleum Hydrocarbons Intrusion 

**Figure 2:**
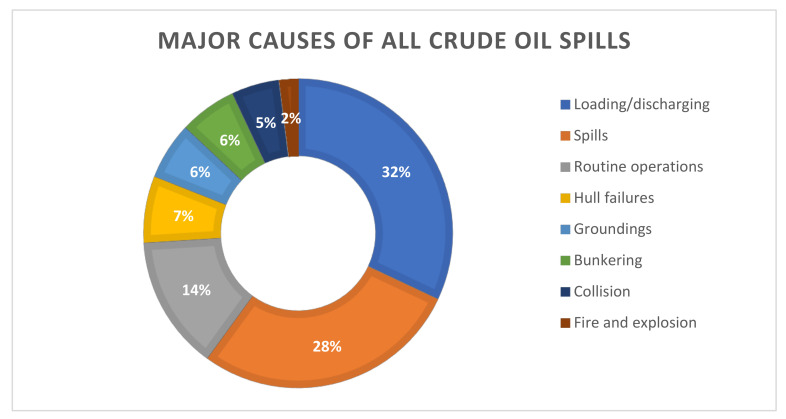
Major causes of all crude oil spills [34].

TPH is released to the environment through oil spill incidents, industrial releases or by-products of private or commercial uses [26]. Crude oil spill in coastal waters is mainly the result of ship operations, tanker accidents, oil exploration and production. The main causes of all the spills are illustrated in Figure 2. In the previous half century, the statistics on the incidence of oil spills have shown a marked downward trend, but still the volume of oil spills is of concern for the environment. More than 7 million tons of hydrocarbon oil from over 140 major spills have been released into the environment [30]. The estimated amount of petroleum hydrocarbon oil lost from tanker discharges alone in 2020 was around 1000 tons [31]. That is the same amount as in the year 2012 and 2019. The list of global oil spills and current spills reveals more than 200 of these incidences in last 50 years on both the offshore and inland waters [32]. In addition to the occurrence of anthropogenic oil spills, millions of tons of petroleum enter the marine environment every year from natural seepages [33].

### 1.3. Properties of Crude Oil

Crude oil is primarily a natural, sticky and flammable liquid. The crude oils vary greatly in chemical composition. It is usually dark brown or black (though it may be yellow or green in color). From an engineering point of view, crude oils are usually classified according to their sources, gravity of the American Petroleum Institute (API) and amount of Sulphur (S). Crude oil is considered “light” when its density is low and “heavy” when it is dense. Crude oils with relatively low sulphur content are called “mild” crudes, while those containing significant amounts of sulphur are called “acid” crudes. Crude oil is a blend of various organic substances, mostly hydrocarbons, organic compound [35]. Petroleum components are divided into four main groups according to their different solubility’s in organic solvents [36,37]. The chemical composition of the crude oil contains the following four main compounds saturated, aromatics, resins and asphaltenes [38]. This is also named SARA [39,40]. Saturated hydrocarbons are regular and branched alkanes with C_n_H_2n+2_ (aliphatic) structure. It contains cyclic alkanes (chain lengths of 1 to 40 or more carbons). Saturated hydrocarbons are the most prevalent constituents of crude oil. Aromatic hydrocarbons are aromatic monocyclic compounds (benzene, toluene, xylene, etc.) and PAHs (naphthalene, anthracene, phenanthrene, etc.). Resins include nitrogen, sulphur, and oxygen-containing polar compounds (for example, pyridine and thiophene). Finally, asphaltenes are poorly polymerized compounds and of high molecular weight. Asphaltenes are poorly characterized hydrocarbons, metals such as nickel, vanadium, and iron also relate to asphalt.

### 1.4. Toxicity of Petroleum Hydrocarbons

**Figure 3:**
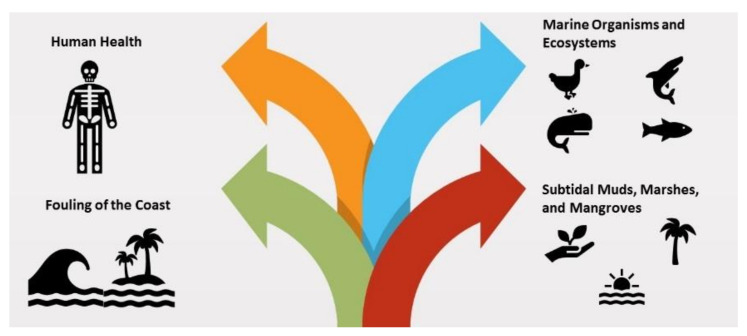
Few impacts of oil spills in the marine environment.

Many factors affect the health effects of exposure to TPH. This involve the form of organic compounds in the TPH, the duration of exposure and the number of chemical substances in contact. Figure 3 illustrate few impacted areas due to petroleum oil spills in the marine environment.

#### 1.4.1. Effects on Marine Organisms

**Figure 4:**
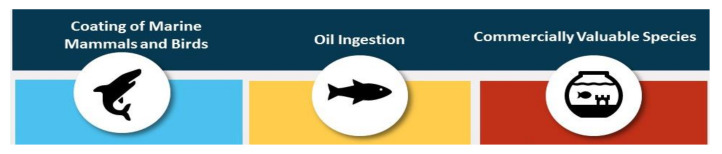
Oil spill in marine environment and ways to affect organisms.

The petroleum hydrocarbons oil spill disaster has an impact on the marine environment and ecosystem [41,42]. As TPH is discharged directly into water bodies by oil spills, petroleum hydrocarbons float on the surface of water and establish thin oily layer. Figure 4 illustrates a few ways a petroleum oil spill can affect organisms in the marine environment.

In situations where the exposure fills the organism’s body with petroleum hydrocarbon oil, direct toxicity is attained and death by smothering takes place [43]. The shallow coral reefs are significant habitat that has been affected by petroleum hydrocarbon oil spills. Coral damage and death following petroleum hydrocarbon oil exposure have been seen extensively [9]. The species have decreased resistance to other environmental stresses, such as variations in temperature, infectious diseases and other pollutants because of petroleum oil that covers the mammals and birds [43]. 

Seabirds are particularly vulnerable because oil contact inhibits the ability to fly. The resulting intake of infected food, inhalation, and repeated encounters with the interface of the oil water result in severe personal poisoning with high mortality rates [9]. Ingested or dissolved oil in the body via membranes, e.g., gill surfaces cause direct lethal toxicity, sublethal effects and marine organisms reproductive failure [43]. Turtles trapped in oil spills are exposed to prolonged physical contact with both floating oils, largely petroleum-saturated respiration air, and the ingestion of food polluted by oil or tar balls. Old and young tortoises were found to be starving to death, as petroleum hydrocarbons blocked their esophagus [9].

The loss of economic capital due to direct mortality, loss of habitat, and restrictions on harvesting and fisheries closures affects the commercial and aquaculture industries [9]. There are negative effects on marketing of commercially valuable species in the aquaculture industry. Similarly, oil taint makes products not suitable for market. Another problem is high concentrations of petroleum oil chemicals of concern for human health in products make then unacceptable for the market [43].

#### 1.4.2. Impacts on Humans

**Figure 5:**
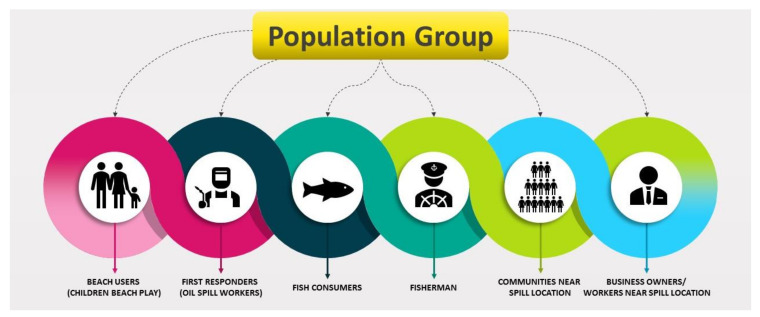
Population of people affected due to oil spill.

First and foremost, in any accident involving petroleum oil spills in the aquatic environment, it is imperative to prevent, if necessary, and reduce the loss of human life and the detrimental effects on human health of the response and clean-up staff and any nearby people and human communities [9,43]. The TPH released on the soil flows into the groundwater through the surface. Some of these chemicals are volatile and evaporate in the air. A few dissolve into the groundwater and move away from the spill area. Most substances bind with soil particles and remain in the soil for a long time, while microbes that are present in the soil break down some hydrocarbons. Secondly, contact may occur via dermal constant contact, inhalation, and ingestion, depending on the properties of the chemical or media (i.e., air, water, soil, food) in which the chemical affects human activity in and around that material [9]. Figure 5 show the population affected by an oil spill accident.

The damage caused by contact to petroleum hydrocarbons can be cancerous, or temporary, or permanently non-cancerous [44]. The numerous chemicals used in dispersants and crude oils poses some documented and alleged health risks [9,45]. Compounds of various fractions of TPH influence the body in different ways. TPH compounds, especially smaller compounds such as benzene, toluene and xylene (which are present in gasoline), can affect the human central nervous system [9,15,46]. Death can occur if exposures are high enough. Breathing toluene at concentrations greater than 100 parts per million (100 ppm) for more than a few hours may induce fatigue, headache, nausea and drowsiness [46]. When the exposure stops, the symptoms will go away. However, if anyone is exposed for a long time, irreversible damage to the central nervous system can result. One TPH compound (n-hexane) can have a distinct effect on the central nervous system, inducing a nervous disease termed “peripheral neuropathy” marked by numbness of the feet and legs and, in extreme cases, paralysis [46]. Swallowing certain petroleum products such as diesel and kerosene causes inflammation of the mouth and stomach, weakness of the central nervous system, trouble coughing, and pneumonia from breathing the fumes of the liquid into the lungs [46]. Compounds in certain TPH fractions may also affect the blood, immune system, liver, spleen, kidneys, developing foetuses, and lungs [46]. Many TPH compounds can be harmful to the skin and eyes. TPH products such as certain mineral oils are not very harmful and are used in food [9,46].

Researchers analyzed the effect of crude oil, dispersants on epithelial cells of human airways and identified similar pathological modes of action for the development of various lung diseases. Their research indicates synergistic effects of crude oil and dispersants important for understanding physical health outcomes and the importance of respiratory safety for particular clean-up crews operating immediately after a spill [45,47]. Other researchers studied the influence of Deep water Horizon (DWH) oil, dispersed mixtures on rodent health in a laboratory setting, with results showing increased influence of the mixture on modifying white blood cells and platelet counts, and affecting liver and kidney function [45,48]. Researchers have reported the acute human health effects among the first responders to the 2007 Hebei Spirit oil spill off the Yellow Sea Coast of South Korea, dumping 12,547 kiloliters of crude oil polluted with 167 km of shoreline and 13,978 hectares of fishery and aquaculture infrastructure, and involving 563,761 clean-up duties [49]. Another study shows that about 442 of the most impacted first responders to the original exposure symptoms were analyzed 1 year later to determine the durability of the toxic effects. Decreased periods of symptoms recorded were eye symptoms (average 9.7 months), headaches (average 8.4 months), skin symptoms (average 8.3 months), neuro-vestibular structures (average 6.9 months), respiratory symptoms (average 2.1 months) and back pain (average 1.8 months) [9,50]. They further reported that it is important to remember that the statistics are merely observational, and some of those who come into contact with volatile compounds during the cleaning operation appear to suffer from these supposed effects after 12 months, with headaches, eye symptoms, neuro-vestibular symptoms, respiratory symptoms, skin symptoms, and back pain in that order.

## 2. Petroleum Hydrocarbon Treatments

Clean-up techniques of hazardous materials are highly influenced by a number of factors such as oil content, oil spill site characteristics and even political considerations [25]. A variety of methods to control oil spills in marine shorelines and freshwater ecosystems have been established but still the problem exists. These methods were closely researched and outlined in several technical documents [25,30,33,43,51,52,53,54]. Floating booms and barriers, oil collection materials, oil collection vessels, absorbing materials, chemical dispersants, surfactants, physical degradation, biodegradation and on-site oil combustion are the most common methods and techniques for oil containment and removal at sea [30]. Clean-up oil is mechanically extracted in significant time using physical techniques. The in situ burning method will contribute to air pollution and, when used with the combustion system, worsen the ambient air quality. Secondly, shoreline vegetation deteriorates as many people manually collect oil and no more than 10–15% of oil recovery take place after a major spill [25,55,56]. The chemical methods of oil removal are faster than physical ones and include toxic chemicals in most situations. Oil spill treatment additives like chemical surfactants are most often harmful rather than oil itself [56,57,58]. Oil spill response workers (OSRWs) are exposed to those operating in the post-emergency process onshore for the purpose of cleaning of oil. OSRWs may be highly exposed to oil spill chemicals by dermal routes and inhalation unless protected and procedures are not followed [59]. Most of the techniques for the recovery or removal of the spilled oil in the water are physical and chemical methods. Oil spill cleaning techniques such as mechanical skimming, sorbents, dispersants, controlled combustion, high-pressure hosing, etc. are quite effective in cleaning up the maximum amount of oil spilled in seawater, but these techniques are not capable of removing emulsified oil left over after physicochemical techniques have been applied. Finally, the complete removal of oil by physical and chemical methods is not achievable and there is remaining residual oil that can be treated with bioremediation. Recent oil spill clean-up methods advantages, limitations and efficiencies are discussed in Table 2.

**Figure 6:**
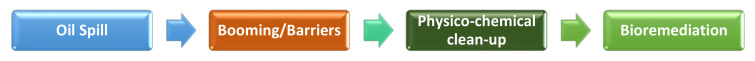
Proposed steps for complete oil spill treatment/clean-up in seawater.

**Figure 7:**
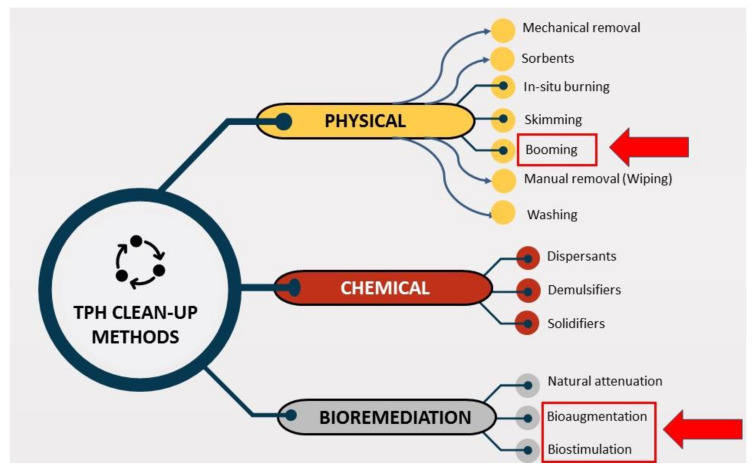
Total petroleum hydrocarbons (TPH) oil spill treatment/clean-up methods.

The treatment steps are discussed in later sections. Figure 6 show the proposed protocol to treat or clean oil spills. Figure 7 show the recent methods used to treat or clean oil spills.

At present, one of the greatest challenges to the application of oil bioremediation in marine water is the lack of guidance about when and how to use this technology [25]. A positive and beneficial aspect is that bioremediation methods may be used in situations where there is less movement of water in the enclosed environment. This form of condition can be created by placing oil containment booms known as booming (Figure 7) on the surface of the water, which are typically used to contain oil spills in moving water and limit movements of moving water with oil spills resulting in an enclosed environment. Floating booms and barriers as the best form of containment for oil spills, followed by oil collection of materials and vessels, have been tested in most cases [30]. The use of oil spill booms as floating barriers should comply with environmental, mechanical and operational constraints. Numerical boom behavior modelling methods may be used to prepare or verify booming strategies that meet these limitations [81]. The residual oil (pollutant) concentration after physicochemical treatment in seawater can be determined by onsite TPH analyzers [82]. Researchers can select an appropriate study from this review article, considering local conditions such as availability of culture of micro-organisms, biostimulants (agro-industrial waste, surfactants etc.), type of TPH pollutants and time to complete bioremediation work. 

In several of the studies mentioned in this review, micro-organisms are either isolated from seawater or enhanced in seawater so that they can be used effectively in their natural environment. Researchers have reported several laboratory scale studies using bioaugmentation (BA), biostimulation (BS) or both methods combined (BA-BS) in aqueous media studies that can be applied on site even after considering the problem due to poor bioavailability of pollutants, protozoan predation or competition from native microbiota, etc. Bioremediation is commonly used as a polishing stage following the application of traditional mechanical clean-up options and is often started from weeks to months following the oil spill [25]. In bioremediation, there is minimal physical damage and short-lived detrimental effects, helping to eliminate certain hazardous elements, a simpler and more rigorous approach, a lower labor intensity and a lower cost [56,75]. Some of the benefits of using bioremediation techniques like BA, BS or both methods combined (BA-BS) are that harmful petroleum hydrocarbons mixtures or combinations are eliminated instead of merely transferred to another nearby environment. Complex processes not applicable in all pollution situations cannot produce substantial short-term outcome and should not be adapted individually to each polluted site as a protective first measures if high concentrations of oil is present [56]. When correctly used in certain oil-contaminated environments, bioremediation has proved to be a cost-effective treatment technique [25,28]. After its successful application in the Exxon Valdez 1989 oil spill, bioremediation has been among the most promising secondary treatment options for oil removal [25,28]. The decision to bioremediate a site depends on the objectives and on all factors, which are present that influence its performance, including clean-up, rejuvenation and habitat preservation.

## 3. Bioremediation

Bioremediation is a process using naturally occurring species to break down hazardous substances into less harmful or non-toxic substances [83]. All substances in nature end up breaking down or decay or transforms into less toxic compounds. In order to obtain energy for their growth, micro-organisms break down many organic compounds in the environment. Bioremediation is also used to reduce pollutant impacts using micro-organisms in the polluted environment. The main reason for clean-up of oil spills is that the toxic and/or hazardous components are reduced or eliminated, allowing flora and fauna to occupy the food chain including single-cell organisms. Since its successful application following the 1989 Exxon Valdez spill, bioremediation has become one of the most promising secondary oil removal treatment solutions [25,84]. While today’s popular chemical dispersants eliminate other harmful aspects of the substance, the toxicity of the spill remains a concern in the area and is sometimes aggravated through adding as dispersants chemicals. The purpose of bioremediation is transform toxic substances to non-toxic substances, such as carbon dioxide, water and fatty acids thereby completely removing petroleum hydrocarbons from the affected environment and returning the affected oil spill zone to its original conditions [25]. The advantage of bioremediation is that the end product is carbon dioxide, water and fatty acids breakdown of hydrocarbons [22,83]. The biological process is an alternate method to eliminate toxins, since this procedure does not cause adverse environmental effects. 

Petroleum hydrocarbons may be used by bacteria [10,33,52], yeasts [11,85,86], fungi [33,87] and algae [78,88]. The regulation of the bioremediation cycle is a difficult process with multiple optimization variables. The key aspect is the energy required for cell growth depending on the metabolic rate of the micro-organism [89]. Cell growth depends on the type of substrates available and consumed by micro-organism. There are basically three types of substrate: primary organic, in which contaminant is considered the main substrate and from this micro-organism consumes energy for further replication. If the pollutant is used as the main substrate and this energy is used to multiply into more cells, it is known as the primary substrate. The second type is secondary organic, where contaminant is known as the secondary substrate, is metabolized by enzymes and helps cells to draw energy. The microbes working in bioremediation with the presence of carbon produces enzymes [8,22]. These enzymes facilitate to break the bonds of hydrocarbons. Various enzymes are used to make this process possible because the metabolism pathways for hydrocarbon reductions are different [22]. It is very important to correctly choose micro-organism based on the enzyme it creates, since this helps to break the hydrocarbons bond. There are different rates of biodegradation of various petroleum hydrocarbon products. It depends, however, on the amount of time required for microbial activity breaking down the hydrocarbons. Therefore, as enzymes help to metabolize [8] and extract energy from the pollutant, the pollutant is known as a secondary substrate. The third type is co-metabolism, while cell energy is obtained from other transformable compounds that are oxidized to sustain microbial growth. In co-metabolism other compounds are oxidized to support microbial growth and energy from other transformable compounds is consumed. Co-metabolism tends to occur when the enzyme formed by the organism can catalyze the degradation of its growth-substrate to generate energy and the carbon from it is also capable of degrading additional compounds [22]. The benefit of co-metabolic bioremediation is also that pollutants can be degraded to trace concentrations, since the microbes in this technique are not reliant on carbon or energy pollutants [90].

Micro-organisms need nutrients (for example nitrogen, phosphate and other trace elements), carbon and energy to survive, as with all living organisms. The rate of biodegradation action depends on the growth conditions of microbes such as nutrient and substratum bioavailability, oxygen availability, electron acceptors, temperature, pH, salinity and pressure [35]. Microorganisms may lack enough nutrients (such as nitrogen, phosphorous, potassium, sulfur, or trace elements) to use the chemical as a source of food. When we compare the elemental composition of petroleum hydrocarbons and micro-organisms, we find that petrochemical residues are not “balance nutritional” for micro-organisms (Table 3) [83]. Biostimulants help to provide the deficit nutrients. Table 3 illustrate the necessary macro-nutrients and Table 4 show micronutrients for a cell microbial metabolism. The effectiveness of bioremediation has been affected by many factors, the most significant being the site’s type of bacteria, the oil and its environment’s physical and chemical conditions. This involves effective bioremediation:

(a) The oiled material is still in contact with nutrients; and
(b) The nutrient concentrations are adequate to help during the cleaning process the optimal growth rate of the oil degrading bacteria [65,74].

## 4. Bioremediation Methods

**Figure 8:**
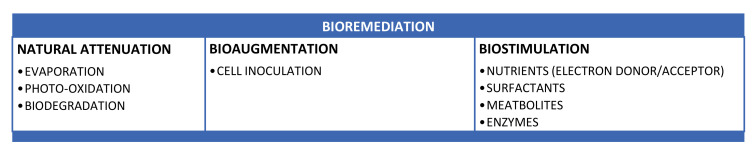
Different types of bioremediation techniques.

Biodegradation is an especially important process for the removal from the atmosphere of non-volatile oil components. Potential bacteria, fungi and algae present in the water steadily break down certain TPH fractions through natural attenuation. That may take months or years to degrade a large proportion of oil that is deposited in the sediments in marine and/or freshwater environments. This is a relatively slow process. Hence, other techniques are used to enhance the bioremediation process. The bioremediation process is enhanced by methods such as bioaugmentation and biostimulation. Bioaugmentation (BA) adds to the indigenous microbial population known oil-degrading microbes and biostimulation (BS) stimulates the growth of indigenous microbes by adding nutrients, electron donors, electron acceptors and other growth enhancing co-substrates and/or environmental changes in conditions (for example, chemical surfactants, biosurfactants etc.) [25]. Natural attenuation (NA) or natural recovery is essentially an option without intervention that allows the removal and natural deterioration of petroleum hydrocarbon oil. In the early stages of oil spills, evaporation of volatile compounds is the most critical method for natural cleaning and the removal of lighter weight components in petroleum hydrocarbon oil. Up to 50% of the more toxic, lighter oil weight components can evaporate within the first 12 h after the oil spill, depending on the composition of the oil spill [25]. Sunlight reacts with oil components by photo-oxidation [8,9,30,43]. Photo-oxidation allows more complicated compounds to degrade into simpler compounds that are typically lighter and more water soluble, so that they can be further extracted by other methods. Various kinds of micro-organism are widely distributed in nature that can oxidize petroleum hydrocarbons [25,33]. For instance, *Actinobacteria* have recently been known viable for hydrocarbon biodegradation analyses due to their high metabolic capabilities. Two properties in particular are of interest in this case; the first is number and variety of degradative pathways for hydrocarbons, and second the development of secondary metabolites such as biosurfactants and siderophores. These properties enable actinobacteria to function under a wide range of environmental conditions and, by secreting metabolites, modify or even alter local conditions [92]. Figure 8 illustrate the types of bioremediation.

**Figure 9:**
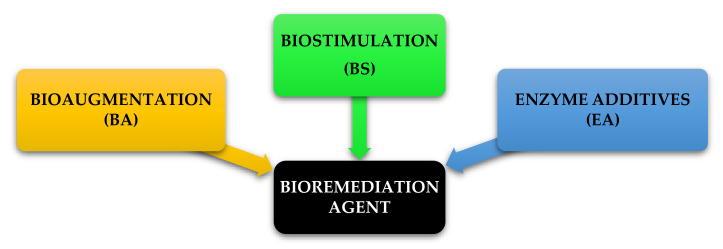
Bioremediation agents under National Oil and Hazardous Substance Pollution Contingency Plan (NCP), USA.

The product schedule of the National Oil and Hazardous Substance Pollution Contingency Plan (NCP), USA, lists dispersants, biological remediators, surface washing agents and various oil spill control agents [93]. All of these are divided into three categories and are illustrated in Figure 9. The first category BA is a method of bioremediation using non-native bacteria. The primary concern with these kinds of products is that introducing foreign species into a given ecosystem unpredictable and future problems may be caused that may be noticeable for some time, although it is useful in controlled/contained environments. The second type of BS consists of some agents that still supply nutrient substrates in the spill area to sustain indigenous microorganisms. BA and BS types are considered to be unsuitable for use in open-water environments [25]. This limitation is due to the inability to hold inoculated micro-organisms culture and nutrients with hydrocarbon pollutants that can be overcome by implementing the proposed method of floating oil containment booms/barriers as proposed in this study. The third type, enzyme additives (EA), is a first reaction system of soil, water and closed environment rejuvenation for open water, intertidal zones, sensitive estuary habitants. Bioremediation experience EA type on the ground has developed in recent years as the technology protocols have dramatically progressed. It provides broad application for oil spillage responses under temperature conditions as low as 28 °F in natural, brackish or marine environments [74]. In addition, bioremediation may be used in some oil-contaminated areas as a proven alternative treatment method. Normally, after conventional mechanical clean-ups it is used as a polishing method. It takes weeks to months to undertake the clean-up. Bioremediation can be very cost-efficient if done correctly, although a detailed economic analysis has not been carried out to date [65]. Bioremediation of polluted hydrocarbon sites can be carried out using BA, BS or both together as BA-BS. 

### 4.1. Bioaugmentation

The process of bioaugmentation is “oil-degrading bacteria are added to supplement the existing microbial population” [65,74]. Bioremediation activities aim to increase the degradation rates that are naturally present by adding exogenous micro-organisms (BA). Bioaugmentation is known as a ‘polishing-up’ or ‘finishing’ process because the impact of fresh oil spill is too slow to turn to less harmful components because the concentration of fresh spilled oil is initially very high. When non-native micro-organisms are exposed to hazardous oil spills, in order to avoid adverse effects to the toxicity of the spill, they seek to release an appropriate amount of biosurfactant and separate from the spill. Petroleum hydrocarbons degrading bacteria (both indigenous and non-indigenous) use intracellular enzymes that allow the bacteria to transform the petroleum hydrocarbons into yet another food source. Oil-degrading microbes produced on a petroleum hydrocarbon-containing culture medium are concentrated microbial agents. The micro-organisms can in some cases be colonized at the site of a spill in bioreactors. Such form of agent is intended to supply the affected region with a substantial oil degrading microbial inoculum, thereby increasing the population that degrades oil down to a point that the spilled oil is used as the main energy source. Case studies included in this review show a good percentage of hydrocarbon degradation by BA, BS or BA-BS in the aqueous medium. The experiments mentioned below in this review were carried out under certain conditions of pH, salinity, temperature, selected micro-organisms as a consortium and oxygen intake. Bioaugmentation techniques are applied for the bioremediation of crude oil, TPH and associated petroleum products in polluted water. Table 5 illustrates a few selected studies for petroleum hydrocarbon degradation using only bioaugmentation.

From the above Table 5 we can see that researchers have used single strain and consortium micro-organisms to degrade petroleum hydrocarbons using the bioaugmentation method. Most of the studies are performed using a consortium micro-organism. In the above studies discussed in Table 5, micro-organisms have been isolated from the polluted site, such as seawater, soil, etc. *Pseudomonas aeruginosa* and *Bacillus subtilis* genera are usually used for bioaugmentation by researchers. Researchers took different concentrations of petroleum hydrocarbons in the biodegradability assay. Petroleum hydrocarbons in the studies were crude oil, diesel, kerosene, gasoline, petroleum, lubricating oil, etc. The range of different concentrations of petroleum hydrocarbons in the biodegradability assay ranged from 0.5% to 5% in all the above studies mentioned in Table 5. The above studies were conducted either in culture medium or seawater. Bioaugmentation-based micro-organisms have been successful in completely degrading petroleum hydrocarbons in some studies and degraded some of the selected components in a few studies. From the above listed studies in the Table 5, maximum degradation efficiency up to 5% (*v/v*) concentration of petroleum hydrocarbons in aqueous medium is observed. 

It took the consortium micro-organisms 7 days to degrade 85% of crude oil at a concentration of 1% *v/v* and the consortium used in this study consisted of *Betaproteobacteria* (47.4%), *Gammaproteobacteria* (51.1%), *Bacillus subtilis* (51.1%) [96]. In a similar study, a consortium of *Bacillus algicol (003-Phe1), Rhodococcus soli (102-Na5), Isoptericolachiayiensis (103-Na4),* and *Pseudoalteromonas agar-Ivorans (SDRB-Py1)* degraded more than 85% of crude oil with a concentration of 1% *v/v* [101]. In another study, a consortium consisting of *Acinetobacter, Pseudomonas, Gordonia, Rhodococcus, Cobetia, Halomonas, Alcanivorax, Marinobacter* and *Microbacterium* took 7 days to degrade 82% of crude oil at a concentration of 1% *v/v* [97]. Researchers observed 81.45% degradation for 1% *v/v* crude oil with the consortium consisting of *Paraburkholderia* sp.*, Alloprevotella tannerae, Paraburkholderiatropica, Ralstonia* sp.*, Paraburkholderiafungorum, Rhodococcus* sp.*, Brevundimonas_diminuta, Lactobacillus* sp.*, Acidocella* sp. and the fungus of *Scedosporiumboydii* [102]. The similar crude oil degradation study was successful with 95% degradation in 20 days using single strain *Alcanivoraxborkumensis SK2* [104]. With respect to diesel, 87% of diesel at a concentration of 2% *v/v* was degraded in 20 days by *Pseudomonas aeruginosa* and *Bacillus subtilis* [99]. The micro-algae *Scenedesmus obliquus GH2* can be used to create an artificial bacteria–microalgae consortium to degrade crude oil [78,105]. Regarding microalgae, *Chlorella vulgaris* degraded 94% of crude oil having 20 g/L concentration in water [103]. A similar study of biodegradation of crude oil was examined by [106], using algae *Chlorella vulgaris* and *Scenedesmus obliquus*. These authors found that both algae are cultured heterotrophically by crude oil as the sole source of carbon and can effectively degrade crude oil when incubated with low crude oil concentrations.

The enhanced bacteria need time to adapt to the fresh available petroleum hydrocarbon oil, environmental temperature, pH and nutrients, but other environmental factors may cause adverse conditions that prevent the disintegration of the oil [22]. These factors along with the unpredictable timescales of their phase of acclimation are partly responsible for the uncertainty associated with the first response clean-up procedure of the form bioremediation BA. The movement of water leads to a totally inefficient dilution of the water, which does not generate adequate biosurfactants, metabolites and enzymes for the destruction of the hydrocarbon molecular structure. A positive and beneficial aspect is that this BA form can be used where very minimal movement of water occurs in the enclosed environment as proposed in this review with floating booms/barriers as an oil containment bioreactor basin [74].

### 4.2. Biostimulation

In many situations, certain environmental conditions can be modified to enhance the process of biodegradation [83]. The process of biostimulation “in which nutrients, or other growth-enhancing, substances, are added to stimulate the growth of indigenous oil degraders” [65]. Bioremediation activities aim to increase the degradation rates by stimulating native micro-organisms (biostimulation (BS)) with nutrients, electron acceptors, electron donors, biosurfactants, metabolites, enzymes etc. Besides the risk of the spill and the perceived ability to compete with already acclimated native bacteria, indigenous bacteria are also more competitive [74]. Therefore, biostimulation has more benefit than bioaugmentation. In certain cases, nutrients are essential components of the effective biodegradation of contaminants, including nitrogen, iron and phosphorus. Some of those nutrients may become an inhibiting factor affecting the biodegradation process. Researchers have mostly used fertilizers as biostimulants. This is because it has N, P, and K. Carbon comes from organic sources (petroleum hydrocarbons), water supplies with hydrogen and oxygen. In marine and freshwater environments, crude oil spills and the effluent from petroleum refineries cause dramatic increases in carbon levels and decreases in nitrogen and phosphorus levels that may affect the process of biodegradation [38,65]. Nitrogen and phosphorus are low in aquatic ecosystems and wetlands cannot provide nutrients due to the high demands on plant nutrients. The introduction of nutrients is, therefore, necessary to facilitate the biodegradation of pollutants. Similarly, nitrogen sources should be considered [13]. For certain situations, nitrogen, phosphorus and iron are important nutrients for a successful process of biodegradation. The most popular additives that promote bacterial growth in the bacterial population are phosphate and nitrate salts. Higher temperatures, (NH_4_)_2_SO_4_ and K_2_HPO_4_ also improve the growth of micro-organisms [19,107]. According to some research into biostimulation of existing oil degraders, there were no lasting gain effects with the introduction of petroleum hydrocarbon oil degrading bacteria [74]. On the other hand, researchers have studied the same problem at lab scale and published promising results, which can be used as a base study for on-site applications to clean-up petroleum hydrocarbon oil spills. 

Biostimulation alone is mostly practiced in soil remediation [108,109,110,111]. Indigenous micro-organisms remain deprived of nutrients in this natural environment. The supply of nutrients to these micro-organisms allows them to degrade the pollutants by carrying out anabolism and catabolism. In a spill area containing toxic oil, nutrients or fertilizers can be difficult to use to promote the development of a crude oil-eating microbial population. The toxicity of the oil initially weakens and/or kills several species native to the spill area. Due to the oil’s toxicity, nutrients are usually prevented from stimulating the remaining indigenous microbes. Where there is no tidal flush and the spilled oil area has reduced toxicity to the degree that indigenous bacteria can be retained (floating booms/barriers as oil containment bioreactor basin), the bioremediation category BS can be used effectively [74].

### 4.3. Bioaugmentation-Biostimulation

Researchers have combined biostimulation and bioaugmentation to predict outcomes when both methods are used together. Such studies have been performed either in seawater or culture medium. Table 6 illustrates a few selected BA-BS studies for degradation of petroleum hydrocarbons.

After looking at the effects of bioaugmentation and biostimulation separately, researchers combined bioaugmentation and biostimulation and obtained better results in a few experiments. From Table 6 it can be concluded that researchers used single strains, and mainly consortia, in studies involving BA-BS. Second, the researchers used stimulants containing predominantly N and P. Third, BA-BS together have demonstrated greater efficiency in degrading petroleum hydrocarbons. Table 6 show that researchers have studied many different combinations of single or consortium micro-organisms with biostimulators like fertilizers, mineral nutrients, chitin and chitosan flakes produced from shrimp waste, corn-steep-liquor, solid-waste-dates, and other materials containing N, K and P. Good results are achieved with corn-steep-liquor, solid-waste-dates, corn-steep-liquor and other materials containing N, K and P. Researchers have achieved 97% degradation efficiency for 0.5% *w/v* crude oil in 28 days by using single strain bacteria *Pseudomonas* and solid-waste-dates as biostimulants [13]. Another related work obtained the 91% degradation by simply changing biostimulant to corn-steep-liquor [13]. The degradation efficiency depends upon the type of TPH pollutant to be degraded. 

Light crude oil degrades more easily and faster than heavy crude oil [22]. Arabian light crude oil (1000 ppm) polluted seawater was degraded by single strain *Alcanivoraxborkumensis SK2* assisted with KH_2_PO_4_ 0.077 g/ L, NH_4_Cl 0.2 g/L and NaNO_3_ 0.1 g/L in 20 days. Similarly, 10% *v/v* crude oil (Escravos light) was degraded 94.4% by *Aspergillus niger* and *Pseudomonas aeruginosa* assisted with (NPK 15:15:15) in 98 weeks (56 days). Regarding diesel, almost complete degradation was archived within 7 days using *Proteobacteria* assisted with surfactant and biosurfactant [7,116].

### 4.4. Natural Attenuation versus Bioaugmentation versus Biostimulation versus Bioaugmentation-Biostimulation

Natural attenuation refers to processes that naturally transform pollutants to less harmful forms or immobilize pollutants so that they are less of a threat to the environment. Bioaugmentation and biostimulation will not be undertaken in natural attenuation. Pollution and natural attenuation of petroleum hydrocarbons needs strategies for remediation of polluted areas. Simultaneous experiments of NA, BA, BS, and BA-BS have been carried out by researchers to compare the methods for the same petroleum hydrocarbon. Table 7 show a few selected bioremediation outcome studies compared with NA, BA, BS, and BA-BS.

From Table 7, it can be concluded that BA, BS and BA-BS provide more degradation efficiency. BA, BS and BA-BS experiments have shown positive results in comparison to natural attenuation. Degradation efficiency of some studies using BA-BS is more than twice the percentage of natural attenuation [13]. This pattern is the same for all research in BSM, MSM, and seawater. It indicates that degradation performance increases with the modification of conditions such as BA, BS and BA-BS. If optimal conditions prevail, this efficiency may increase and take even less time than previous studies. The degradation time and efficiencies in the above Table 7 varies with the type (light or heavy crude oil), concentration of pollutant, and micro-organisms inoculated assisted with stimulators. 

Researchers used BA and BS to treat crude oil polluted water using mixed microbial cultures *Aspergillus niger* and *Pseudomonas aeruginosa*. Four samples of oil hydrocarbon-polluted water were monitored for eight weeks using the following bioremediation techniques: control (nutrient-free), A (nutrient NPK 15:15:15), B (nutrient-plus aeration), and C (nutrient-free, aeration, and agitation). For the A, B and C samples respectively, reductions of TPH were 92.3%, 93.6% and 94.4%. The pH was within the range of 6–9 for all samples [18]. Similar studies have been performed in the Gulf of Taranto (Italy) for the actual oil spill sample. In April 2012, more than 20 metric tons of cargo fuel oil was discharged by an unknown source, covering an area of about 800 m^2^. Approximately, 250 L of oil-polluted seawater was collected and transported to a laboratory immediately after 24 h of the spillage. The research was conducted in a tank of size 62 cm × 40 cm × 30 cm each and for 14 days. In order to compare NA, BS and BA-BS methods, 200 L of oily seawater was distributed in separate microcosms: (1) NA; (2) BS (nutrients: KH_2_PO_4_ 0.077 g/L, NH_4_Cl 0.2 g/L and NaNO_3_ 0.1 g/L); (3) BA-BS (consortium: *Alcanivorax borkumensis, Alcanivorax dieselolei, Marinobacter hydrocarbonoclasticus, Cycloclasticus* sp. 78-ME and *Thalassolituus oleivorans)* and nutrients as in the BS treatment; (4) washing agent with oily-seawater and nutrients as in the BS treatment. The degradation efficiencies for NA, BS and BA-BS was 32 ± 3.2%, 73 ± 2.4%, and 79 ± 3.2% respectively [98]. Another study in seawater was performed using tank experiments. In this study, seawater was lifted by direct pipeline from the Messina Strait. During the entire experimental phase, the seawater was aerated and continuously stirred. The seawater was held at 18 ± 2 °C. The experiments were performed in an 11,250 L (5000 cm × 150 cm × 150 cm) rectangular tank filled with 10,000 L of seawater. The experiments were performed in three separate tanks. BS (crude oil and inorganic nutrients); BA1 (*A. borkumensis SK2T*); BA2 (*A. borkuminsis SK2T + T. oleivorans MIL-1B*). In all experiments, sterile Arabic light crude oil (10 mg/L) and inorganic nutrients were supplemented with seawater. The inorganic nutrients (sterile) were (final concentrations: KH_2_PO_4_ 0.077 g/L, NH_4_Cl 0.2 g/L and NaNO_3_ 0.1 g/L). The biodegradation study found that the degradation of BA1 was the highest (95%) compared to BS (80%) and BA2 (70%) [104]. These studies are yet to be evaluated under real on-site conditions as indicated and proposed in this review by floating oil containment booms as a bioreactor basin.

## 5. Conclusions

Physical and chemical oil spill clean-up methods are ineffective at completely cleaning up the petroleum hydrocarbons of oil spilled in seawater and are not capable of removing emulsified oil left over after physico-chemical techniques have been applied. The complete removal of petroleum hydrocarbons oil by physical and chemical methods is not achievable and there is remaining residual oil that can be treated with bioremediation. The lack of guidance on the use of this technology is now one of the greatest challenges for petroleum hydrocarbons oil bioremediation in marine waters. The possibility of bioremediation methods is a good and beneficial factor, where there is less water movement in the area surrounded by water. It can be achieved by floating oil containment booms, which are generally used to cover flowing water oil spills and to limit water movement through the oil spills that generate a confined area. Bioremediation can be used in some petroleum hydrocarbon polluted areas as a proven alternative clean-up/treatment method in combination with floating oil containment booms to enclose the petroleum hydrocarbon polluted areas and act like a bioreactor basin. Several of the studies mentioned in the article are laboratory-based studies that have the potential to be applied in the field (on-site) and are still to be evaluated. This is an untapped area and has scope in the future. In many of the studies mentioned in this article, micro-organisms are either isolated from seawater or enhanced in seawater so that they can be used effectively in their native environment (on-site). The biostimulants mentioned as low-cost substrates have a large potential and have been proven in laboratory-based studies that can be used in petroleum hydrocarbon remediation. BS and BA-BS techniques would lead to the use of agro-industrial waste and to sustainable treatment. At the same time, two problems are resolved: the pollution problem of oil spills treatment and the utilization of agro-industrial waste. The disadvantages and difficulties that may be encountered in the use of these studies are outlined in the future scope section of the article. It is difficult to mention all data from a study in a table format. The outcomes of the studies are, therefore, shown for the primary reference for bioremediation using BA,BS and BA-BS. Researchers may refer to the requirements of the particular study referred to in this review paper based on their suitability and use either BA, BS and BA-BS as a viable bioremediation technique in combination with a booming technique to enclose the oil spill as in the bioreactor basin. Case studies reviewed in this paper may help environmental researchers adopt an appropriate method for the bioremediation of a petroleum hydrocarbon pollutant in seawater, estuaries, and beaches for the cleaning of emulsified oil left over by using BA, BS and BA-BS methods.

## 6. Future Scope

Due to the conditions discussed in this review paper, bioremediation (BA and BS type) of open flowing water is not deemed appropriate. There is scope here to identify the method or technology to be used (BA and BS type) for flowing water sources such as seawater and rivers. There are a few drawbacks of BA, BS and BA-BS as applied to moving water bodies such as seawater and rivers. Some of these drawbacks can be overcome by booms/barriers method as discussed in this review. The drawbacks are listed below:

- Nutrients are instantly diluted in nearly background quantities which do not bind in fresh or weathered hydrocarbons/oil, if nutrients are added to flowing water. It is often difficult to collect or add nutrient substrates to oil spills, in windy and otherwise adverse weather conditions, which cause waves.
- In an oil spill pollution environment containing toxic oil, it is difficult to use additional nutrients for micro-organisms which eat hydrocarbons. From the beginning, the toxicity of oil damages and/or kills several species native to the spill area. The nutrients are typically prohibited from improving the other indigenous microbes because of the toxicity of oil.
- However, it is a major problem to supply adequate amounts of deficit nutrients i.e., nitrogen and phosphorous, in an effort to increase the population of petroleum hydrocarbons degrading bacteria without raising the concentrations of nitrogen and phosphorous to the amount that it is harmful to marine water life. The method of improving indigenous organisms using nutrients and fertilizers is uncertain and sometimes takes a long time, with the hope that there will be sufficient secretion of biosurfactants, metabolites and enzymes to catalyze the bioremediation process. The greatest challenge to the respondent is to create the right conditions for optimal biodegradation, i.e., to keep sufficient nitrogen and phosphorus concentrations in seawater always.
- Normally, after conventional mechanical clean-ups, bioremediation is used as a polishing method. It takes weeks to complete the clean-up, which is quite slow. This can be very cost-efficient if done correctly, although a detailed economic analysis has not been carried out to date.

## Figures and Tables

**Table 1:** Elemental composition of crude oil.

Sr.No.	Elements	Percentage (%)
1.	Carbon	85–90
2.	Hydrogen	10–14
3.	Sulfur	0.2–3
4.	Nitrogen	<0.1–2
5.	Oxygen	1–1.5
6.	Metals *	<1

* Hg, Au, Cu, Al, Ca, Co, K, Mg, Si, Sr, Mo, Ti, Mn, Li, Se, Rb, Ag, Ba, Pb, As, Cd, Cr, Fe, Ni, V, Zn.

**Table 2:** Comparison of oil spill clean-up methods.

	Clean-Up Methods	Advantages	Disadvantages	Maximum Clean-up Efficiency	Application	References
**Physical**	Sorbents	Recovery of oil which prevents wastage and more pollution	After the oil absorption, it is difficult to retrieve sorbent materials; Become heavier and sink, difficult to retrieve and sink to cover benthic organisms	90%	Most effective in small oil spills or leftover traces of a larger spill	[60,61,62,63,64]
	Washing	Remove the trapped and weathered oil from machinery-inaccessible areas.	Organisms that fall into the direct spray zone are likely to be harmed by hot water (170 °C).	-	Mechanical removal methods such as booms and skimmers are inaccessible or unavailable for oil clean-up.	[25,30,54,60]
	In-situ burning	Where it is difficult to deploy other methods	Burning sites pollute the air and can impact ecosystem both onshore and offshore; Residue from in situ burning reaches coastlines or in worse condition, sink to cover benthic organisms; Fire-resistant booms are high in cost, difficult in towing due to size and heavy weight.	98%	Arctic or sub-Arctic environments (remoteness and sea ice formations); the oil slick thickness was also adequate for the combustion to continue; Seawater was calm and oil slick was not located in vulnerable areas, equipment or facilities	[25,30,60,65,66]
	Skimming	Recover oil without changing its physical or chemical properties by suction and adhesion	Surface conditions: wind and waves disperse oil in the water (rough seas can stop skimmers from effective functioning); To get the equipment operating and to the site on time (as the spilled oil will quickly spread over quite a few km^2^)	95%	Less movement of water	[25,30,60,64,65,66]
	Booming	Light weight, limited storage space, non-corrosive and fast processing, highly efficient where water movement is lower	Low stability in strong winds and currents (current velocity more than 0.4 m/s, wind velocity greater than 5.5 m/s or height of waves more than 1 m)	-	Oil is at one spot; spillage is reachable within a few hours, or the spill area becomes too vast to handle.	[60,67,68,69]
	Manual removal (Wiping)	Economically viable (unskilled personnel can be employed with minimum training)	Labour-intensive and time-consuming	15%	Shorelines oil slick clean-up	[60,67,68]
**Chemical**	Dispersants	Break up oil slicks to avoid the coastlines and vulnerable habitats covering vast volumes of oil; Not much manpower required (cheaper than physical methods)	Poisoning fish, corals, and other marine species	90%	If the spilled oil cannot be stopped by booms and spread over large areas; May be used in rough seas, slowing emulsion formation from oil water, speeding up natural biodegradation	[60,67,68,69,70,71]
	Solidifiers	Convert oil spill into solid or semi-solid materials; Not much manpower required (cheaper than physical methods)	Oil recovery not possible (oil recovery with high viscosity not effective)	-	May be used in rough seas	[63,68,69,72]
	Demulsifiers	Impede the spread and pollution of oil in nearby areas; Not much manpower required (cheaper than physical methods)	The gelatine used may pose a risk of entangling or suffocating the aquatic animals	-	May be used in rough seas	[41,60,68,69]
**Bioremediation**	Natural attenuation	Most cost-effective and sustainable methods; Not much manpower required	Quite time-consuming and unreliable	Yet to be evaluated	Areas close to the shoreline	[41,43,73,74,75,76,77,78,79,80]
	Bioaugmentation		Quite time consuming			
	Biostimulation		Quite time consuming			

**Table 3:** Comparison for elemental composition of a microbial cell with petroleum crude oil [91].

Elements	Microbial Cell Composition (%)	Crude Oil Composition (%)
Carbon	50	85–90
Nitrogen	14	<0.1–2
Oxygen	20	1–1.5
Hydrogen	8	10–14
Phosphorous	3	-
Sulphur	1	0.2–3
Potassium	1	-
Sodium	1	-
Calcium	0.5	-
Magnesium	0.5	-
Chloride	0.5	-
Iron	0.2	-
All others	0.3	<1

**Table 4:** Micro-nutrients for cell growth and their cellular functions.

Micro Nutrients	Cellular Functions
Cobalt	Vitamin B12; transcarboxylase (propionic acid bacteria)
Copper	Respiration (cytochrome c oxidase); Photosynthesis (plastocyanin, some superoxide dismutases)
Manganese	Acts as activator of various enzymes; occurs in some superoxide dismutases and in the photolytic (water-splitting) enzyme in oxygenic phototrophs(photosystem-II)
Molybdenum	Present in some flavin containing enzymes, nitrogenase, nitrate reductase, sulphide oxidase, some formate dehydrogenases.
Nickel	Present in most hydrogenase enzymes; coenzyme of methanogenes; carbon monoxide dehydrogenase; urease
Selenium	Occurs in formate dehydrogenase; certain hydrogenases: amino acid selenocysteine
Tungsten	In some formate dehydrogenases; oxotransferases of hyperthermo-philes
Vanadium	Vanadium nitrogenase; bromoperoxidase.
Zinc	In carbonic anhydrase; alcohol dehydrogenase; RNA and DNA polymerase; many DNA-binding proteins.

**Table 5:** List of selected studies for degradation of petroleum hydrocarbons using bioaugmentation (BA).

References	Pollutant	Micro-Organisms	Degraded Efficiencies	Time
[94]	0.5% (*v/v*) petroleum oil	*Pseudomonas, Rhodococcus and Acinetobacter.*	66%	15 days
[95]	1% (*v/v*) crude oil	*Bacillus sp.,* *Corynebacterium sp.,* *Pseudomonas sp.,* *Pseudomonas sp.*	77%	25 days
[96]	1% (*v/v*) crude oil	*Betaproteobacteria,* *Gammaproteobacteria,* *Bacillus subtilis*	85.01%	7 days
[97]	1% (*v/v*) crude oil	*Acinetobacter,* *Pseudomonas,* *Gordonia,* *Rhodococcus,* *Cobetia,* *Halomonas,* *Alcanivorax,* *Marinobacter,* *Microbacterium*	82%	7 days
[98]	2% (*v/v*) Cargo fuel	*Alcanivoraxborkumensis,* *Alcanivoraxdieselolei, Marinobacterhydrocarbonoclasticus,* *Cycloclasticus sp.,* *Thalassolituusoleivorans*	79 ± 3.2%	14 days
[99]	2% (*v/v*) diesel	*Pseudomonas aeruginosa,* *Bacillus subtilis*	87%	20 days
[100]	5% (*v/v*) kerosene	*Citrobactersedlakii,* *Entrobacterhormeachei,* *Entrobacter cloacae*	69%	7 days
[101]	1% (*v/v*) crude oil	*Bacillus algicola (003-Phe1),* *Rhodococcus soli (102-Na5), Isoptericolachiayiensis (103-Na4), Pseudoalteromonas agar-* *ivorans (SDRB-Py1)*	>85%	14 days
[102]	1% (*v/v*) crude oil	*Paraburkholderia sp.,* *Alloprevotellatannerae,* *Paraburkholderiatropica,* *Ralstonia sp.,* *Paraburkholderiafungorum,* *Rhodococcus sp.,* *Brevundimonas_diminuta,* *Lactobacillus sp.,* *Acidocella sp.,* *Fungus Scedosporiumboydii*	81.45%	7 days
[103]	20 (g/L) crude oil/water	*Chlorella vulgaris*	94%	14 days
[104]	10 mg/L crude oil polluted seawater	*Alcanivoraxborkumensis SK2*	95%	20 days

**Table 6:** List of selected studies for degradation of petroleum hydrocarbons using bioaugmentation–biostimulation (BA-BS).

References	Pollutant	Micro-Organisms	Degraded (%)	Time	Stimulator
[112]	0.5% (*v/v*) Crude oil polluted seawater	*Rhodococcuscorynebacterioides*	60%	15 days	Chitin and Chitosan flakes (shrimp wastes)
[113]	0.1% (*v/v*) weathered crude oil in seawater	*Thalassolituus,* *Alcanivorax,* *Cycloclasticus*	85%	30 days	Nutrients (20 mg/L NH_4_ NO_3_ and 10 mg/L KH_2_PO_4_)
[114]	nC15–nC35 (TPH = 10 g/L)	*Pseudomonas aeruginosa Asph2*	80%	30 days	Corn-steep-liquor
[18]	10% (*v/v*) Crude oil	*Aspergillus niger,* *Pseudomonas aeruginosa*	94.4%	8 week	NPK 15:15:15
[115]	1000 ppm polluted seawater	*Alcanivoraxborkumensis SK2*	95%	20 days	KH_2_PO_4_ 0.077 g/L, NH_4_Cl 0.2 g/L and NaNO_3_ 0.1 g/L
[13]	0.5% (*w/v*) crude oil	*Pseudomonas*	97%	28 days	Solid-waste-dates
[13]	0.5% (*w/v*) crude oil	*Pseudomonas*	91%	28 days	Corn-steep-liquor
[98]	2% (*v/v*) Cargo fuel oily seawater	*Alcanivoraxborkumensis, Alcanivoraxdieselolei, Marinobacterhydrocarbonoclasticus,* *Cycloclasticus sp. 78-ME,* *Thalassolituusoleivorans*	73 ± 2.4%	14 days	KH_2_PO_4_ 0.077 g/L, NH_4_Cl 0.2 g/L and NaNO_3_ 0.1 g/L
[101]	1% (*v/v*) Crude oil	*Bacillus algicola (003-Phe1), Rhodococcus soli (102-Na5), Isoptericola chiayiensis (103-Na4),* *Pseudoalteromonas agar-* *ivorans (SDRB-Py1)*	>85%	14 days	Biosurfactant assisted
[7,116]	1% (*v/v*) Diesel oil	*Proteobacteria*	20–99%	7 days	Surfactant (Tween-80), biosurfactant (rhamnolipids)

**Table 7:** Comparison of different bioremediation outcomes on petroleum hydrocarbons.

Medium	Natural Attenuation %	BA %	BS %	BA-BS %	Time	References
Polluted water	50.7	-	94.4	-	8 weeks	[18]
BSM ^#^	38	66	-	91	28 days	[13]
BSM ^#^	38	66	-	97	28 days	[13]
Seawater	32 ± 3.2	-	73 ± 2.4	79 ± 3.2	14 days	[98]
Seawater	-	95	80	-	20 days	[104]
MSM *	-	81.45	-	-	7 days	[102]

^#^ Basal Salt Medium; * Mineral Salt Medium.

## Data Availability

Not applicable.
